# Myocardial Infarction in a Young Adult: A Rare Case of Left Coronary Artery Arising from the Pulmonary Artery

**DOI:** 10.3390/life15091482

**Published:** 2025-09-21

**Authors:** Stefan Veljković, Jovana Lakčević, Ana Peruničić, Armin Šljivo, Miloš Babić, Slobodan Tomić, Jelena Kljajević, Sanja Vučinić, Milovan Bojić, Aleksandra Nikolić

**Affiliations:** 1 Cardiovascular Institute “Dedinje”, 11040 Belgrade, Serbia; jovana.lakcevic@gmail.com (J.L.); anaperunicic@hotmail.com (A.P.); babicmisa@hotmail.com (M.B.); bobantomic99@gmail.com (S.T.); jelenakljajevic@gmail.com (J.K.); sanjakosticvucinic@gmail.com (S.V.); dedinje@ikvbd.com (M.B.); nikolicdrsasa@gmail.com (A.N.); 2 Department for Cardiovascular Surgery, Clinical Center of University of Sarajevo, 71000 Sarajevo, Bosnia and Herzegovina; sljivo95@windowslive.com; 3 Faculty of Medicine, University of Banja Luka, 78000 Banja Luka, Bosnia and Herzegovina; 4 Faculty of Medicine, University of Belgrade, 11000 Belgrade, Serbia

**Keywords:** coronary vessel anomalies, coronary artery disease, myocardial ischemia, heart failure, adult congenital heart disease

## Abstract

Anomalous origin of the Left Coronary Artery from the Pulmonary Artery (ALCAPA), also known as Bland-White-Garland syndrome, is a rare congenital coronary anomaly with an estimated incidence of 1 in 300,000 live births. While commonly diagnosed in infancy, adult presentations are exceedingly rare and pose significant diagnostic challenges. Delayed diagnosis may result in progressive myocardial ischemia, heart failure, arrhythmias, or sudden cardiac death. Surgical correction is the definitive treatment, with the goal of restoring a dual coronary artery system and preventing irreversible myocardial damage. We present the case of a 30-year-old male with a prior history of non–ST-elevation myocardial infarction who was referred for evaluation of exertional angina and symptoms of heart failure. Transthoracic echocardiography revealed a dilated left ventricle with an ejection fraction (LVEF) of 35%. Coronary angiography and cardiac MDCT identified an anomalous origin of the left circumflex artery (LCx) from the right pulmonary artery (RPA) and a coronary–pulmonary artery fistula involving the LAD. The patient underwent successful surgical correction with reimplantation of the LCx into the ascending aorta. Postoperative recovery was uneventful. At 3-month follow-up the patient was symptom-free, though echocardiography revealed persistent LV dilation and reduced LVEF, necessitating continued pharmacologic therapy and monitoring. This case highlights the importance of maintaining a high index of suspicion for ALCAPA in adult patients with unexplained cardiomyopathy or ischemic symptoms. Early diagnosis and surgical intervention remain crucial for improving long-term outcomes and preventing life-threatening complications.

## 1. Introduction

Anomalous Left Coronary Artery from the Pulmonary Artery (ALCAPA), also known as Bland-White-Garland Syndrome, is a rare congenital heart disease characterized by the abnormal origin of the left coronary artery from the pulmonary artery instead of the aorta, leading to reduced myocardial oxygenation and potential clinical manifestations such as angina, heart failure, or sudden cardiac death, typically diagnosed in infancy or childhood [1,2,3].

ALCAPA is an exceptionally rare congenital anomaly, with an estimated incidence of approximately 1 in 300,000 live births and constitutes 0.24% and 0.46% of all congenital cardiac disease [4]. The condition is often fatal in early life if left untreated, as the abnormal origin of the left coronary artery from the low-pressure pulmonary circulation results in insufficient perfusion of the left ventricular myocardium, which leads to left ventricular dysfunction, mitral regurgitation, arrhythmias, or sudden cardiac death [3]. Consequently, without timely surgical intervention, the prognosis in infancy is poor, and only about 10–15% of individuals with undiagnosed or untreated ALCAPA survive into adulthood [1,5,6].

Definitive treatment of ALCAPA is surgical, with the primary goal of re-establishing a dual-coronary artery system to ensure adequate myocardial perfusion. The standard approach is direct reimplantation of the anomalous left coronary artery (LCA) into the aorta, a procedure that restores normal coronary artery anatomy [7]. In cases where direct reimplantation is not feasible due to anatomic considerations or the presence of significant myocardial damage, alternative surgical options may be employed, including the Takeuchi procedure, which creates an intracoronary baffle to redirect blood flow from the pulmonary artery to the aorta, or coronary artery bypass grafting (CABG) to restore coronary perfusion [7]. Early surgical intervention, particularly when performed before the development of irreversible myocardial ischemia and fibrosis, is associated with improved long-term survival rates and functional recovery, including normalization or significant improvement in left ventricular ejection fraction (LVEF) [8]. However, adult survivors of ALCAPA, particularly those with long-standing myocardial damage, require lifelong follow-up for the monitoring of arrhythmias, persistent left ventricular dysfunction, and the potential need for an implantable cardioverter-defibrillator (ICD) in high-risk patients. The risk of sustained ventricular arrhythmias and sudden cardiac death remains elevated in this population, necessitating close cardiovascular surveillance and management [8].

In this case report, we present a unique case of ALCAPA in a young adult patient who presented with symptoms of heart failure and angina and was ultimately diagnosed and treated using a combination of clinical and imaging techniques. The patient underwent successful surgical intervention, and we discuss the challenges and considerations in the management of adult patients with this rare congenital heart disease [1].

## 2. Case Presentation

A 30-year-old male was referred to our institution for further evaluation of exertional angina and symptoms of heart failure. The patient’s medical history was significant for a non–ST-elevation myocardial infarction (NSTEMI) in 2019, for which he underwent coronary angiography at a regional clinical center.

On admission, a 12-lead electrocardiogram (ECG) demonstrated sinus rhythm at a rate of 60 beats per minute, rS complexes in the inferior and precordial leads V1–V4, and frequent premature ventricular contractions.

Laboratory tests revealed mildly elevated high-sensitivity troponin I at 45 ng/L (reference range: <14 ng/L), BNP level at 780 pg/mL (normal < 100 pg/mL), NT-proBNP at 3200 pg/mL (reference < 125 pg/mL), CK-MB at 12 U/L (reference < 7 U/L), CRP at 3.1 mg/L (normal < 5 mg/L). Renal function was preserved, with a serum creatinine of 0.9 mg/dL and an estimated glomerular filtration rate (eGFR) above 90 mL/min/1.73 m^2^. Liver enzymes were within normal limits, including AST (35 U/L) and ALT (30 U/L). Lactate dehydrogenase (LDH) was mildly elevated at 280 U/L (reference 135–225 U/L). Serum electrolytes were within the normal range (sodium 139 mmol/L, potassium 4.1 mmol/L). Glycemic control was adequate, with an HbA1c of 5.4% (reference < 5.7%), and the lipid profile was unremarkable: total cholesterol 170 mg/dL, LDL cholesterol 90 mg/dL, HDL cholesterol 42 mg/dL, and triglycerides 130 mg/dL.

Transthoracic echocardiography revealed a dilated left ventricle with global systolic dysfunction. Specifically, the left ventricular end-diastolic diameter (LVEDD) was measured at 62 mm (normal range: 42–58 mm), and the left ventricular end-systolic diameter (LVESD) was 47 mm (normal range: 25–40 mm). Regional wall motion abnormalities were observed in the basal segments of the interventricular septum, inferior wall, and posterior wall. The LVEF was estimated at 35%. Additionally, the left atrium was mildly dilated, with an anteroposterior diameter of 42 mm (normal range: 19–40 mm).

**Figure 1:**
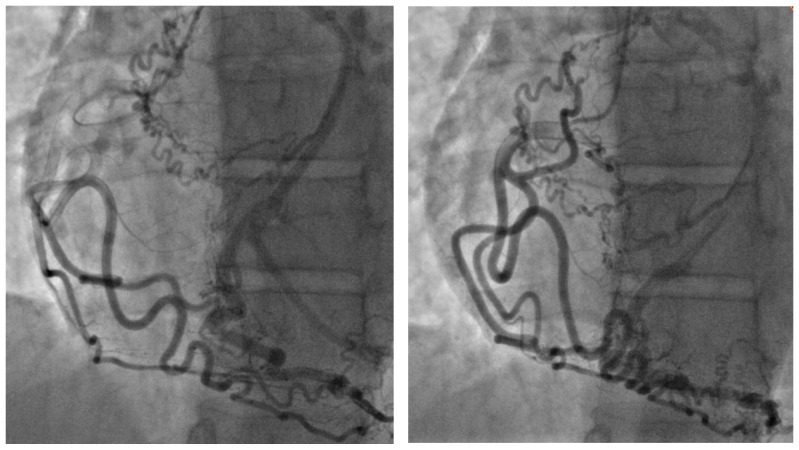
Coronary angiography in the right anterior oblique projection demonstrating the anomalous origin of the left circumflex artery (LCx) from the pulmonary artery (PA). Numerous tortuous collateral vessels arising predominantly from the right coronary artery (RCA) provide retrograde perfusion to the LCx territory. The contrast flow pattern highlights the collateral-dependent circulation and underscores the hemodynamic significance of this anomaly.

**Figure 2:**
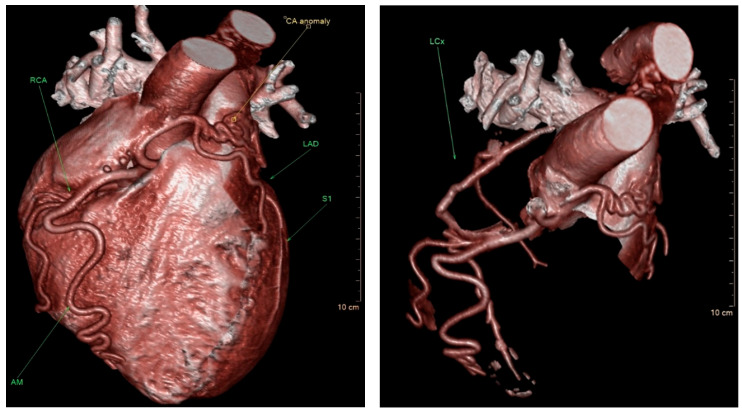
Three-dimensional volume-rendered multidetector computed tomography (MDCT) of the heart demonstrating complex coronary anomalies. Both the right coronary artery (RCA) and the left anterior descending artery (LAD) originate from the right coronary cusp (RCC). A coronary–pulmonary artery fistula is visualized between the LAD and the pulmonary artery (PA). Additionally, the left circumflex artery (LCx) is shown arising anomalously from the right branch of the PA.

The coronary angiographic findings revealed no significant coronary artery stenosis. However, the procedure identified an anomalous and separate origin of the left coronary artery (LCA) from the right coronary cusp (RCC) (Figure 1). Given the patient’s persistent symptoms and the angiographic findings, further evaluation was pursued. Multidetector computed tomography (MDCT) of the heart was performed, which confirmed the diagnosis of ALCAPA. The MDCT demonstrated coronary arteries originating from the RCC, including the left anterior descending coronary artery (LAD) with a coronary–pulmonary artery (PA) fistula, and an anomalous origin of the left circumflex (LCx) coronary artery from the right pulmonary artery (RPA) (Figure 2).

Following a comprehensive evaluation and discussions with the patient about the risks and benefits of various treatment options, surgical correction of the ALCAPA was deemed necessary, given the persistent heart failure symptoms, angina, and severity of the condition, in order to prevent further myocardial ischemia and preserve long-term cardiac function. In October 2022, the patient underwent a successful surgical procedure involving excision of the anomalous LCx and its subsequent reimplantation into the ascending aorta. This procedure was performed through a median sternotomy, with careful dissection of the coronary vessels to ensure adequate revascularization and preservation of myocardial perfusion.

The postoperative course was uneventful, and the patient was monitored in the intensive care unit for several days for hemodynamic stability. By the time of discharge, the patient was clinically stable, with no complications related to the procedure. At a three-month follow-up appointment, the patient reported complete resolution of his symptoms, including heart failure and angina. However, follow-up transthoracic echocardiography revealed persistent left ventricular dilation, with LVEDD of 62 mm, unchanged regional wall motion abnormalities, and a reduced LVEF of 35%. Despite these findings, the patient was clinically asymptomatic and had no further episodes of angina or heart failure.

Given the ongoing evidence of left ventricular dysfunction, the patient is being closely monitored through regular follow-up visits, including serial echocardiograms and clinical assessments, to evaluate any changes in cardiac function or further management needs. The patient has been advised to continue with pharmacologic therapy, including beta-blockers and angiotensin-converting enzyme inhibitors (ACE inhibitors), to manage heart failure and prevent any potential deterioration in cardiac function.

## 3. Discussion

ALCAPA is a rare congenital coronary anomaly, typically diagnosed during infancy due to symptoms of myocardial ischemia or heart failure. In contrast, survival into adulthood is exceedingly uncommon and usually associated with the development of sufficient intercoronary collateral circulation. In adult patients, the diagnosis of ALCAPA remains challenging, often requiring a high index of clinical suspicion in the context of non-specific symptoms such as exertional chest pain, dyspnea, or arrhythmias. This case highlights the diagnostic complexity of ALCAPA in the adult population and underscores the necessity for timely recognition and surgical management to prevent potentially fatal complications such as malignant arrhythmias, left ventricular dysfunction, or sudden cardiac death. Unlike the classical pediatric form, which usually manifests with severe ischemia and heart failure in infancy, and the adult form, which more often presents with exertional angina, arrhythmias, or sudden death, our patient exhibited an exceptionally rare constellation of anomalies, including an anomalous LCx arising from the RPA and a coronary–pulmonary artery fistula originating from the LAD.

The clinical presentation of ALCAPA in adult patients is highly variable, ranging from completely asymptomatic cases discovered incidentally to severe, life-threatening manifestations such as exertional angina, progressive dyspnea, syncope, palpitations due to ventricular arrhythmias, heart failure, or even sudden cardiac death, particularly during physical exertion. The variability in symptomatology is largely influenced by the extent of collateral circulation between the right and left coronary arteries and the degree of myocardial ischemia or infarction. These collaterals partially compensate for the anomalous origin of the left coronary artery, supplying the myocardium and allowing patients to remain asymptomatic or present with milder symptoms for years. However, despite this compensation, the left ventricular myocardium remains vulnerable to chronic subendocardial ischemia, particularly during increased oxygen demand. This persistent ischemia can lead to myocardial fibrosis, left ventricular dysfunction, and predisposition to malignant arrhythmias. A more comprehensive explanation of these mechanisms, including the balance between collateral-dependent perfusion and ongoing ischemic stress, would enhance the readers’ understanding of the adult presentation and the rationale for timely surgical correction [9,10]. Given its rarity and nonspecific clinical presentation in adults, the diagnosis of ALCAPA necessitates a thorough and systematic diagnostic approach. Initial evaluation typically includes a resting ECG, which may show signs of prior infarction or nonspecific ST-T wave changes. Transthoracic echocardiography is often the first imaging modality employed, although it may be limited in sensitivity, particularly in adults. For definitive anatomical delineation, advanced imaging techniques such as MDCT, cardiac magnetic resonance imaging, and invasive coronary angiography are essential. These modalities provide critical information regarding coronary anatomy, myocardial perfusion, and ventricular function, all of which are necessary for preoperative planning [1,2,11,12].

In this case, the patient presented with symptoms of heart failure and angina, which led to the diagnosis of ALCAPA. The MDCT of the heart revealed an anomalous and separate origin of the LCA from the RCC, as well as an anomalous origin of the LCx from the RPA. This diagnosis was confirmed by surgical excision of the anomalous LCx and reimplantation into the ascending aorta [5,6]. Surgical intervention is generally indicated in all adult patients with ALCAPA, regardless of symptomatology, given the inherent risk of catastrophic cardiac events even in seemingly asymptomatic individuals [13,14]. The surgical goal is to establish a two-coronary artery system and restore antegrade flow to the left coronary artery to eliminate myocardial ischemia and reduce left-to-right shunting.

The preferred surgical technique is direct coronary reimplantation, wherein the anomalous left coronary artery is excised from the pulmonary artery and reimplanted into the aorta. This technique may be performed with or without the use of an interposition graft, depending on coronary length and anatomic constraints. In cases where direct reimplantation is not technically feasible—due to anatomic limitations, excessive tension, or coronary size mismatch—an alternative strategy includes CABG in conjunction with surgical closure or ligation of the anomalous ostium in the pulmonary artery to prevent coronary steal [11,12,13,14].

Postoperative outcomes in adults are generally favorable, with improvements noted in left ventricular ejection fraction, regression of ischemic symptoms, and reduced arrhythmic burden. Nonetheless, long-term follow-up with serial imaging and functional assessment is critical, as late complications such as graft failure, progressive mitral regurgitation, or arrhythmias may occur [8,14].

In this case, the patient underwent successful surgical correction of the anomalous origin of the LCx, with intraoperative findings confirming the anomalous origin and adequate revascularization achieved. The postoperative course was uneventful, with no perioperative complications such as arrhythmias, hemodynamic instability, or myocardial injury. The patient was discharged in stable condition on evidence-based pharmacologic therapy, including beta-blockers, ACE inhibitors, and diuretics as indicated for left ventricular dysfunction. At the 3-month follow-up, the patient remained clinically stable and asymptomatic, with no reported exertional angina, dyspnea, or syncope. However, transthoracic echocardiography demonstrated persistent left ventricular enlargement with global hypokinesis and an unchanged wall motion pattern. The LVEF remained reduced at 35%, consistent with chronic systolic dysfunction likely due to irreversible myocardial injury and remodeling from prolonged ischemia prior to surgical intervention.

## 4. Conclusions

ALCAPA is a rare congenital heart disease that can present in adult patients with a wide range of symptoms. A high level of suspicion and a combination of clinical and imaging techniques are necessary for accurate diagnosis and appropriate management. Surgical correction is often recommended for symptomatic adult patients with ALCAP. Consequently, careful longitudinal follow-up is warranted, and in cases of sustained left ventricular dysfunction or arrhythmic events, consideration should be given to ICD therapy as a secondary or even primary preventive strategy.

## Data Availability

The data can be shared up on request.
